# What has biotelemetry ever done for avian translocations?

**DOI:** 10.1186/s40462-022-00359-w

**Published:** 2022-12-07

**Authors:** Simon C. R. Lee, David J. Hodgson, Stuart Bearhop

**Affiliations:** 1 Centre for Ecology and Conservation, University of Exeter, Penryn, UK; 2 Natural England, York, UK

**Keywords:** Conservation translocation, Reintroduction, Biologging, Ecological restoration, Satellite tracking, Remote sensing, Animal behaviour, Movement ecology

## Abstract

Species translocation is a popular approach in contemporary ecological restoration and rewilding. Improving the efficacy of conservation translocation programmes requires a combination of robust data from comparable populations, population viability modelling and post-release monitoring. Biotelemetry is becoming an ever more accessible means to collect some of the high-resolution information on the ecology and behaviour of founding populations that such evaluations require. Here, we review 81 published case studies to consider how this capability could increase the success of avian translocations. We found that 67 translocations favoured traditional radio telemetry, with surveillance focussing mostly on immediate post-release dispersal, survival and breeding attempts. Just 28 projects tracked founder individuals for longer than 1 year and no studies referenced pre-release sampling or planning using biotelemetry. While our review shows that tracking devices have been deployed extensively in translocation projects, its application has been mostly limited to short-term spatial and demographic monitoring. We conclude that biotelemetry is a powerful tool for harnessing a multitude of lifetime eco-behavioural data which can be used to build valuable predictive models and surveillance programmes, but this capability has yet to be fully realised by researchers in avian translocations.

**Supplementary Information:**

The online version contains supplementary material available at 10.1186/s40462-022-00359-w.

## Introduction

Human activities are leading to a global extinction crisis [12], with some 12% of the planet’s c.10,000 bird species potentially being lost forever by the end of the 21st Century [33]. Predatory birds tend to be more sensitive to anthropogenic threats and extinction due to their generally slow life histories and low population densities [29]. Depletion of functions and services provided by these species will impact ecosystems profoundly [15]. While the challenge of reversing worldwide declines is daunting, rebuilding biodiversity at the scale of populations and geographical regions is eminently achievable [20]. Conservation translocations are “the human-mediated movement of living organisms from one area, with release in another” [25]. When properly planned and actioned, conservation translocations are an effective method to reinforce and/or re-establish populations [25]. Contemporary baseline biological data from comparable populations, viability modelling and rigorous post-release monitoring are all features of successful translocation programmes [16, 25].

Although motivations and aims for undertaking conservation translocations vary, they can be generally classified across a spectrum comprising population reintroduction, reinforcement and trophic wilding through to ecological replacement, assisted colonisation and community construction, as detailed in Table 1 [36]. Some authors have posed the idea of de-extinction, the process of resurrecting extinct species, as a means of restoring ecosystems, although its potential to contribute effectively to biodiversity conservation is untested [34].

**Table 1:** Translocation spectrum. Adapted from [36]

Term	Aim of translocation	References
De-extinction	Resurrection (through technological means) and reinstatement of formerly extinct species	Robert et al. [34]
Reintroduction	Re-establishment of population following extinction or extirpation	IUCN [25], Seddon [36]
Reinforcement	Supplementation of an existing population of conspecifics to improve viability through increased recruitment and/or genetic heterogeneity	IUCN [25], Seddon [36]
Trophic wilding	Creation of ecological interactions and/or cascades to promote self-regulating biodiverse ecosystems, including mitigation of biological invasions and improving biotic resistance	Svenning et al. [38], Fortin et al. [19]
Ecological replacement	Replacement of an extinct species with a close relative of the same genus to fill their specific ecological niche	Seddon and Soorae [37], IUCN [25]
Assisted colonisation	Relocation outside of indigenous range to mitigate extinction or perceived anthropogenic threats like climate change and wholesale habitat loss	Carter et al. [10], IUCN [25]
Community construction	Creation of entirely new or novel species assemblages and ecosystems	Seddon [36], Seastedt et al. [35]

Historically many translocations have failed, often quite inexplicably at the time, prompting conservation scientists to call for wider implementation of appropriately planned and funded research and post release monitoring programmes to help improve outcomes and understanding of likely causes of failure [16, 25]. Bubac et al. [8] reviewed translocations published from 2005 to 2016 across a range of taxa, evaluating approaches to post-release monitoring as well as factors that were associated with success or failure [8]. While they found 54% of translocations did succeed, 20.9% and 25.1% of studies reported failure or an unknown outcome respectively, with no evidence that success rates had actually increased over the study period [8]. Amongst other considerations, Bubac et al. [8] stressed the importance of pre-release assessment, including detailed life-history accounts of species, and long-term post-release monitoring to help improve success rates of translocations [8].

Biotelemetry has become an ever more powerful and accessible means to gather much of the detailed information required for the effective evaluation of translocation projects [41]. Tracking technologies now enable ecologists to collect high quality data at unprecedented spatial scales from the comfort of the office or laboratory [30, 42]. Integrating the disciplines of biomechanics and ecology, tri-axial acceleration is especially potent for providing rich data on behaviour and energy expenditure [31, 44]. Furthermore, the longevity of many modern devices facilitate the circannual tracking of individuals over multiple years [27]. Year-round, uninterrupted relocations can help overcome customary seasonal bias in sampling (often focussed on breeding periods) and yields intelligence on vital rates, migration and dispersal, facilitating better understanding of founder population dynamics [28]. Fundamentally, biotelemetry can help bridge the data gap left by traditional monitoring techniques [27]. For example, conventional marking only provides information at the point of capture and recovery (or re-sighting), while tracking data can depict a near-continuous picture over all or substantial parts of an animal’s life [26]. Such knowledge is of critical importance for planning and managing successful conservation translocations [30].

Modern biotelemetry could play a significant role in pre-release and post-release monitoring studies, not least because it is capable of detecting more subtle parameters such as individual decision-making and personality, which command a superior understanding and management of the likely dynamics being played out in translocated populations [26]. Therefore, biotelemetry is potentially an important means for enhancing the outcomes of all types of conservation translocation [9, 14]. In this paper, we explore the extent to which avian translocations have utilised biotelemetry through a review of published case studies. We discuss our findings and highlight some practical applications of biotelemetry within the framework of a hypothetical translocation project.

## Review of biotelemetry in avian translocations

### Methods

A comprehensive search for articles on avian translocations published between 2005 and 2021 was conducted using Thomson Reuters Web of Science and Google Scholar databases. A publication year threshold of 2005 was applied to reduce bias from historic deployment of heavier tags on larger species. We identified full articles (reviews were excluded) using the topic search terms and Boolean Operators translocat* OR reintro* OR re-intro* AND bird OR avian OR raptor from journal categories of ornithology, ecology, biodiversity conservation, environmental sciences, zoology and biology. Of the 325 relevant papers generated, we excluded translocations that did not report the use of biotelemetry, producing 81 papers for detailed case study review. For each retained paper, we documented the main tracking technologies deployed and key demographic and ecological parameters likely to be important for translocation assessment and monitoring. For further background on these factors, we refer the reader to an overview of biotelemetry hardware and use in ecology study (Additional file 1)provided in the Supporting Information. The selected parameters and their recorded incidence are summarised in Table 2, with full review data (Additional file 2) also provided in the Supporting Information.

**Table 2:** Summary of case study review parameters and results

Demographic and ecological parameters	Recorded frequency	Tracking technology deployed	Recorded frequency
Breeding attempts	42	GLS tags	1
Breeding dispersal	1	GPS tags	14
Habitat selection	20	GSM tags	4
Home range	28	Radio tags	67
Initial dispersal	60	Satellite PTT tags	18
Migration routes	6	Onboard sensors	0
Mortality causes	42		
Natal dispersal	5	*Other parameters*	
Population range	8	Family	29
Productivity	24	Species	54
Social behaviour	9	Tag/harness effects	3
Survival	61	Whole-year monitoring	28

### Results

Case studies covered 54 species with a size (mass) range of 27 g–16 kg across 29 families, providing both taxonomic breadth and a cross-section of typical tracking technologies available to researchers during the study period.

#### Tracking technologies

Of the 81 translocations reviewed (noting that some projects deployed multiple types of devices), the majority (67) favoured traditional radio transmitters, manually triangulating bird locations via hand-held receivers. Of the remainder, 18 projects deployed satellite Platform Terminal Transmitters (PTT’s) and four used tags that transferred data via the Global System for Mobile communications (GSM). Just 14 studies utilised Global Positioning System (GPS) enabled devices, while only one project fitted Global Location Sensor (GLS) loggers. Notably, no case studies referenced deployment of tags with additional on-board sensors such as accelerometers.

#### Demographic and ecological parameters

For the majority of studies, capturing demographic responses to translocation was the primary driver of monitoring effort. For example, the majority of translocations documented survival (61), and over half identified causes of mortality (42) and first breeding attempts (42). Importantly, just one quarter measured key data such as productivity (24). While 28 projects monitored founders for longer than 1 year, only a handful of studies reported longer term breeding information such as natal and breeding dispersal distances (five and one, respectively). As for broader ecological monitoring, a large proportion of studies assessed immediate dispersal from the release location (60), although other spatial-based parameters such as home ranges (28) and habitat selection (20) were examined to a lesser degree. Just eight translocations were able to monitor at sufficient scales to record population ranges (eight) and only six gathered data on migration routes. Group interactions were poorly monitored with just nine translocations recording any kind of social behaviour. Three papers evaluated tag or harness effects and no studies referenced pre-release data collection using biotelemetry, despite a small number of translocations being experimental or exploratory in nature.

## Discussion

**Fig. 1:**
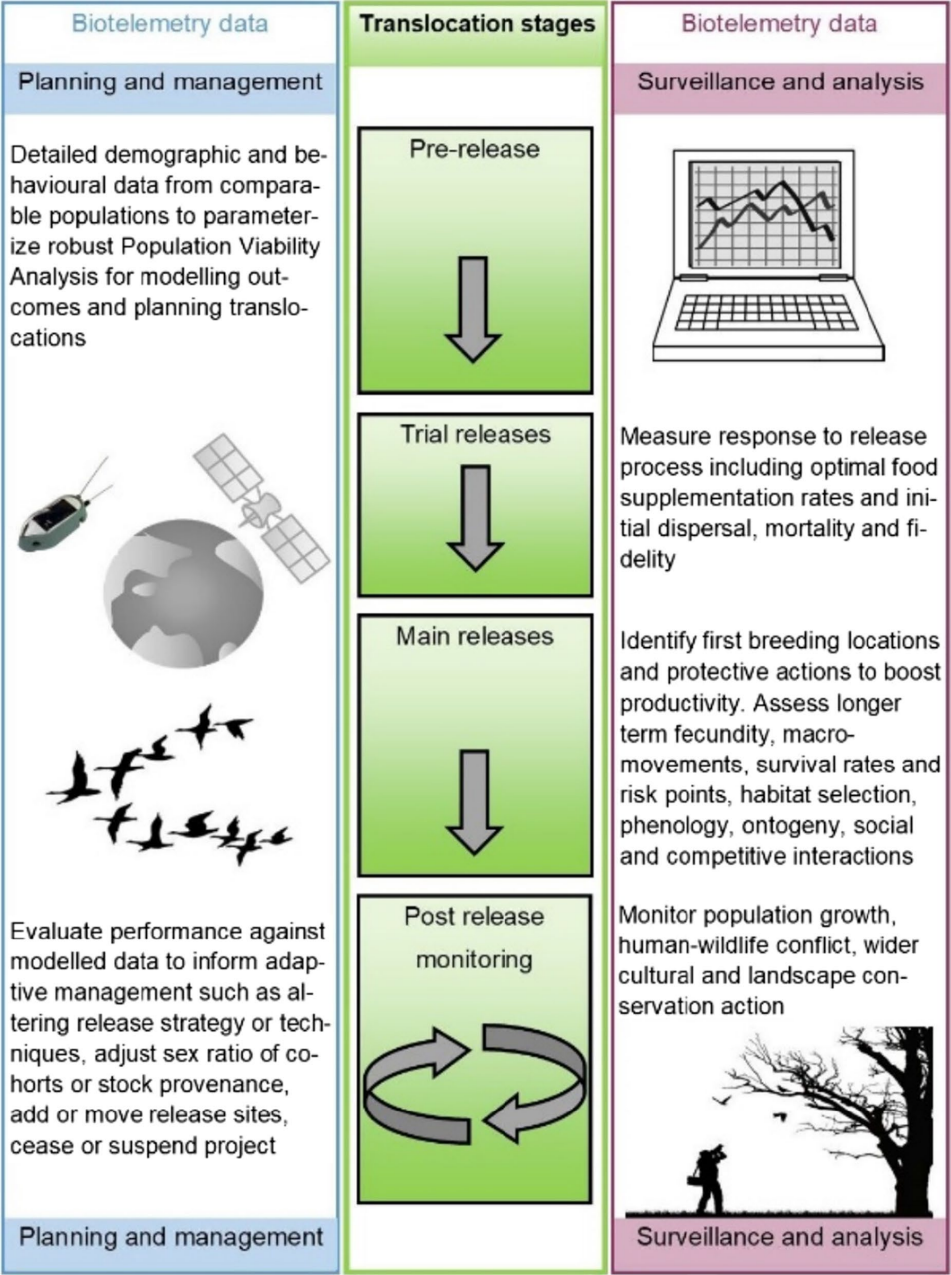
How biotelemetry can inform stages of a hypothetical translocation

Biotelemetry is helping to transform ecological and behavioural research, and with at least 81 published cases since 2005, our review shows clearly that tracking devices have been widely deployed in avian translocations [9]. However, we have also identified potential applications relevant to avian translocations that were unused or under-exploited by our case studies. In this section we illustrate the untapped benefits of this technology to researchers and practitioners using the framework of a hypothetical translocation project. Our framework comprises the following phases: (a) pre-release for modelling and planning; (b) trial release(s) to test logistics and initial founder responses; (c) further releases for population establishment; (d) ongoing post-release monitoring for adaptive management, engagement and long-term performance. Figure 1 provides an illustrative summary of this framework.

It is important to note that there is an inherent risk to each study animal whenever a tracking device is attached [5]. Therefore, the ethics of biotelemetry is a balance of costs and benefits where researchers must offset potential negative effects of capture and tagging by maximising the benefits of their study [26]. Continued miniaturisation and longevity of tags and refinement of attachment methods is a strong driver for manufacturers and conservation scientists [39]. In light of the above, we suggest that our framework provides a useful template and set of considerations for researchers and practitioners to maximise the benefits of biotelemetry to improve the overall outcomes of conservation translocations.

### Pre-release

Holistic understanding of founder ecology and behaviour prior to embarking on a release programme is key to predicting the future dynamics of a translocated population [3, 8]. Modelling parameters like vital rates, habitat preferences, mortality risk points, natal and breeding dispersal and migration patterns can provide essential insights into a new population’s likely viability under different management and timescale scenarios [16].

Modern biotelemetry provides the opportunity to harvest such essential data, continuously and remotely on many individuals over multiple years [26, 42]. Yet, our review identified traditional hand-held radio telemetry as the technology of choice which only confers limited gains over non-telemetered data collection methods [1, 24]. Of additional value to translocations is the ability to estimate measures of key activities such as foraging distances and success rates, including adult provisioning of young at the nest. Analysis of high-resolution GPS and acceleration data can help determine individual hunting ‘effort’ for given seasons, habitats and/or geographic areas [44]. Such data are invaluable for modelling the likely carrying capacity of comparable receptor habitats and landscapes, providing an insight into the likely rate and pattern of expansion of translocated populations, but rarely feature in the translocations we investigated.

Although conducting specific pre-translocation research may not always be feasible (perhaps due to the lack of wild subjects and/or financial constraints), with increasing efficacy of remote tracking and interest in conservation translocations for more widespread species, biotelemetry has a much greater role to play in data collection for planning purposes [8].

High quality information and analyses that informs project design, monitoring and adaptive management can only improve overall likelihood of translocation success [25]. Population Viability Analysis (PVA) is an established and critical tool in probabilistic modelling of the persistence of translocated populations under different release strategies [1]. PVAs rely on realistic parameter estimation of population demography, ecology and behaviour, and the better the data, the more helpful the model [40]. Likewise, selection of appropriate tracking technology is key to providing the depth and duration of the ecological information required [42]. Contemporary biotelemetry clearly has a significant role in mining such data.

### Trial releases

Before embarking on a main programme, scientifically robust trials or experimental releases can help remove potentially flawed prior assumptions of founder ecology, a factor which has frequently hampered the effectiveness of translocations historically [2]. Whether a trial, or simply the first year of longer planned releases, comprehensive monitoring at this stage is critical for understanding founders’ initial physical and behavioural response to the overall release process and thus informative to the expected progression (or not) and strategy for the main stages of a translocation [16].

It is common practice (especially with raptors) to provide supplementary food for founders over extended periods at the release site or within the wider landscape, not only to promote optimal long-term health but also to serve as a population management tool, for example, to encourage fidelity to a specific area [17]. Measuring return rates to areas of interest will provide invaluable information on the early health and foraging fitness of translocated individuals, which may also be insightful for determining longer-term drivers and patterns of dispersal, migration and demography [17, 27]. And it is here where modern GPS and smart sensor enabled tracking devices are likely to be at the forefront of capturing such valuable information [26, 27]. While we have noted that a small number were experimental or exploratory in nature, none of our case studies reported use of biotelemetry explicitly for dedicated trials or modelling purposes in preparation for main translocation phases.

### Main releases

New tracking technologies can provide remote and enhanced detection of early breeding attempts, especially for secretive individuals or species that nest in isolated locations [7, 43]. This information facilitates additional protection or management measures which may be crucial for boosting fecundity of embryonic populations [27]. Monitoring seasonal and age-related demographic responses, such as natal and breeding dispersal patterns, are also key to forecasting and managing how young populations will establish, spread and sustain themselves [16]. More subtle parameters like social and competitive interactions can also influence overall population performance, all of which can be better understood though multi-individual biotelemetric methods [26, 27]. Without fitting devices equipped with on-board sensors (let alone to multiple animals), there is limited opportunity to analyse individual behaviour in any depth, nor start to build a picture of community level interactions [42].

While around quarter of the case studies we reviewed captured initial home ranges of translocated birds, most were too brief to measure longer-term population responses such as reproductive performance, seasonal changes in habitat requirements and range development. Similarly, the lack of multi-year monitoring in turn prevented data collection on migratory routes, barriers, stopovers and strategies and any potential for studying individual ontogeny was completely foregone. While some case studies considered habitat use immediately after release, very few performed real analysis or characterisation of wider landscape use and or indeed interpolation with remote sensed environmental data. Arguably such knowledge may be considered peripheral over core metrics of dispersal and survival of founders. However, robust measures of whole-life demographic, spatial use, habitat preferences and behavioural ecology are essential for modelling and managing persistence of translocated populations [28].

### Post-release monitoring

Post-release monitoring is catch-all term usually describing observations and data collection conducted during translocations, although here we extend the meaning to include a period of surveillance beyond the release phases [8]. PRM provides information on population establishment, not only in evaluating future viability but also in gauging the relative success or otherwise of the overall translocation programme [4]. Without a comprehensive monitoring programme, which biotelemetry can significantly enhance, judgement on the status of translocations is impossible or potentially flawed [18]. Crucially, appropriate post-release monitoring provides the metrics and feedback mechanisms that inform the adaptive management of translocations that will aid ultimate success [25]. These actions could include altering release strategy or techniques, adjusting the sex ratio of release cohorts, moving or adding sites, changing or mixing donor stock and even suspending or ceasing translocations [23].

Most translocations take place within human modified landscapes, and their success is often reliant on societal acceptance and support, best achieved through properly planned public and stakeholder engagement programmes [32]. The ability of biotelemetry to remotely capture movement and behaviour for ecological study is an equally potent means to engage people in the detail and drama of animals’ lives, especially via well publicised social media and web platforms [6, 21].

## Conclusion and implications for practice

Has biotelemetry helped to improve the overall success of translocations? From the evidence we found, it is hard to draw any firm conclusions. While our case studies show that biotelemetry has been extensively used in translocation projects, its application has been mostly limited to short-term monitoring of dispersal and survival of founders. Reviewed projects mostly favoured radio-based telemetry, perhaps due to lower unit costs of tags and the limited availability of suitable satellite/GSM/GPS devices historically [24]. However, apparent savings of radio technology need to be balanced with potentially higher overall monitoring costs as a result of additional labour-intensive fieldwork [13].

While flagging potential risks of deployment, we know that contemporary tracking devices can provide a multitude of long-term eco-behavioural measures from which researchers can build predictive models and post-release monitoring programmes, yet this capability awaits more widespread adoption by conservation managers [26, 42]. With such capacity also comes increased complexity, dictating an ever-greater need for specialist analytical skills to determine meaningful biological signal from the derived datasets [22]. Fully harnessing the ability of biotelemetry will require prudent selection of tracking technologies alongside considered project design and detailed knowledge of study subjects to best achieve the desired project goals [24, 27]. Therefore, research and monitoring needs to be set within a robust structure along the lines of our suggested monitoring framework for optimal performance of release programmes [16].

There is perhaps a negative popular view of the value of conservation translocations arising from historic low success rates and perceived high costs of these interventions [11]. Our review highlights that biotelemetry is an increasingly powerful tool to study animal movement and behaviour, and if targeted at the right questions, can only be beneficial to the outcomes of translocation programmes, which in turn may help recast their societal reputation.

## Supplementary Information

**Additional file 1.** Biotelemetry hardware and use in ecological study.

**Additional file 2**. Case studies full record

## Data Availability

All data generated or analysed during this study are included in this published article and its supplementary information files.
