# Correction: What has biotelemetry ever done for avian translocations?

**DOI:** 10.1186/s40462-023-00370-9

**Published:** 2023-02-02

**Authors:** Simon C. R. Lee, David J. Hodgson, Stuart Bearhop

**Affiliations:** 1 Centre for Ecology and Conservation, University of Exeter, Penryn, UK; 2 Natural England, Sterling House, Dix’s Field, Exeter, UK

**Correction to: Movement Ecology (2022) 10:57** https://doi.org/10.1186/s40462-022-00359-w

Following publication of the original article [1], the authors identified an error in the affiliations assignment: the second and third author, David J. Hodgson and Stuart Bearhop, should have had the Centre for Ecology and Conservation, University of Exeter (affiliation 1) assigned as affiliation, instead of Natural England (affiliation 2).

This error is corrected in the author list of this Correction article and the original article [1] has been updated.
