# Supplementary material for: What has biotelemetry ever done for avian translocations?
Source: Mov Ecol. 2022 Dec 7;10:57. doi: 10.1186/s40462-022-00359-w (PMC9727958; doi:10.1186/s40462-022-00359-w)
Supplement: Supplementary file 1 — Additional file 1. Biotelemetry hardware and use in ecological study. [file 40462_2022_359_MOESM1_ESM.docx]

**Supplement to: What has biotelemetry ever done for avian translocations?**

**Simon C. R. Lee, David J. Hodgson & Stuart Bearhop**

**Overview of biotelemetry hardware and use in ecological study**

This document compliments the main text by providing some additional technical information that the reader may find informative. We draw your attention to the allied term of ‘biologging’, which typically refers to archival devices requiring recovery to extract stored data (Whitford and Klimley 2019). We do not cover the respective pros and cons of devices and systems as these are discussed elsewhere, for example, Tomkiewicz et al (2010) and Lopez-Lopez (2016) provide excellent synopses of the array of technologies available for animal tracking (Tomkiewicz et al. 2010; Lopez-Lopez 2016). Instead, we summarise the essentials of tracking technologies (Table 1), consider some of the key operational and ethical considerations of deployment and their application in broader ecological study.

**Biotelemetry hardware and operational considerations**

*Geolocation methods*

Historically, radio telemetry has been the technology of choice in avian studies, typically operated through use of hand-held VHF/UHF receivers, calculating location coordinates by triangulation (Kenward 2000). Early satellite tags or Platform Terminal Transmitters (PTTs) utilise the Argos constellation to estimate geolocations via Doppler shift analysis of the PTT uplink frequency (Hofman et al. 2019). Although strictly speaking biologgers, Geolocators or Global Location Sensing (GLS) tags are popular in studies of seabirds and small birds (Lopez-Lopez 2016). Light level geolocation uses solar irradiance to establish geographical location, obtained by calculating day length to specify latitude and the time of solar noon for longitude (Lopez-Lopez 2016). More recently, use of the Global Positioning System (GPS) satellite-based navigation system provides geolocations in three dimensions (i.e. latitude, longitude, altitude) plus a precise timestamp (Tomkiewicz et al. 2010). Most tags on the market today employ GPS, although other operational satellite systems such as GLONASS, Galileo Compass and the developing ICARUS project are potentially available for use in avian biotelemetry (Tomkiewicz et al. 2010).

**Table 1. Overview of common avian tracking technologies**

| **Tracking technology** | **Description** | **References** |
| --- | --- | --- |
| **Location method**  Radio (VHF/UHF/RFID)  Doppler | 2D, short range, location by triangulation or directly by receiver  2D, calculated from transmission frequency to ARGOS satellites  3D, ubiquitous satellite-based navigation system  2D, estimates location via day length and time of solar noon | (Lopez-Lopez 2016)  (Hofman et al. 2019) |
| GPS  GLS (geolocator) |  | (Tomkiewicz et al. 2010)  (Lopez-Lopez 2016) |
| **Data retrieval**  Satellite (e.g. Argos, Iridium)  Radio – manual, local  Radio - automated, global | Arrays of global satellite systems used for a variety of data transfer  RFID/VHF receivers and modems for local data transmission.  Networks of receivers with automated download such as Motus | (Lopez-Lopez 2016; Hofman et al. 2019)  (Taylor et al. 2017) |
| GSM/GPRS | Digital mobile telephone system, worldwide coverage, bi-directional | (Lopez-Lopez 2016; Tomkiewicz et al. 2010) |
| **On-board sensors**  Accelerometer  Gyroscope  Magnetometer  Temperature | Acceleration and orientation data over time, typically in three axes  Provides tilt angle (rotation) data, typically in three dimensions  Detects earth’s magnetic field, acts as an electronic compass  Measures device CPU, animal body and ambient temperature  Digital sensor to determine atmospheric pressure and altitude  Detects whether bird is submerged in seawater or airborne  Multi-directional, identifying social and interspecific interactions | (Whitford and Klimley  2019; Wilson, Shepard, and Liebsch 2008) |
| Barometric pressure  Salt-water switches  Proximity sensors |  | (Mattern et al. 2015)  (Williams et al. 2020) |

*Data transfer*

Miniaturization of electronic components has led to much wider use of automated radio-based data transfer systems from transmitters (Tomkiewicz et al. 2010). For example, Radio Frequency Identification (RFID) technology employs Passive Integrated Transponder (PIT) tags to record when a known individual passes close to readers (Williams et al. 2020; Tomkiewicz et al. 2010). The new ‘Motus’ multi-partner collaboration extends VHF technology to worldwide arrays of receiving towers operating on a single frequency and centralised database (Taylor et al. 2017). Bi-directional data transfer can also be facilitated through remotely placed VHF beacons and narrow-band Frequency Modulation (FM) modems (Tomkiewicz et al. 2010).

Global satellite communication systems, notably Argos, Iridium and Globalstar, are widely used in tracking studies to transfer packets of geolocation and sensor data stored in the memory of tags (Tomkiewicz et al. 2010). With an expanding cellular network, Global System for Mobile communications (GSM) and Short Message Service (SMS) offers energy efficient transfer for smaller data sets over much of Europe and Asia (Tomkiewicz et al. 2010). Although GSM service is currently limited in parts of Africa, Australia and North America, data is nonetheless stored onboard for subsequent download in areas with good coverage (Tomkiewicz et al. 2010). Larger data sets require the General Packet Radio Service (GPRS) in addition to GSM, when available (Lopez-Lopez 2016). GSM and some satellite systems also provide bi-directional communication allowing tag settings to be adjusted during deployment (Tomkiewicz et al. 2010; Lopez-Lopez 2016). In GSM, satellite and Motus systems, recovered data is processed centrally by service providers and accessed by the end user via a base station computer or web interface (Hofman et al. 2019).

*On-board sensors*

The sensors described below now feature commonly as integral components of many modern tracking devices (Kays et al. 2015). Accelerometers measure acceleration over time, usually in three axes (X, Y, Z) and provide data on fine scale body movements (Lopez-Lopez 2016). Gyroscopes record rotation, while magnetometers detect the earth’s magnetic field, acting as an electronic compass (Lopez-Lopez 2016). Digital barometric pressure sensors determine atmospheric pressure and altitude (Whitford and Klimley 2019; Wilson, Shepard, and Liebsch 2008).

The combination the above sensors affords use of Inertial Navigation System (INS) technology, which can calculate continuously the geolocation, orientation and velocity of the device between GPS fixes, enabling behavioural analyses. Temperature sensors measure internal device and external ambient temperature (Whitford and Klimley 2019; Wilson, Shepard, and Liebsch 2008). Salt-water switches register whether the device is submerged in sea water or airborne (or on land) and proximity sensors detect inter- and intra-specific encounters (Mattern et al. 2015; Williams et al. 2020).

*Attachment methods*

Many attachment techniques have been developed for avian research, although only certain methods are suitable for particular taxa, dictated by the dimensions, mass, body design and flight mode of the subject and the desired deployment period (Kenward 2000; Boitani and Fuller 2000). For longer-term studies, tracking devices are mounted externally, commonly with backpack or leg-loop style harnesses made from hypo-allergenic materials such as Teflon tape and Elastane (Kenward 2000). Short-term deployments typically affix devices with adhesive or via tail feather-mounted methods, sometimes in combination with sutures, threads, Velcro or cable ties (Kenward 2000; Boitani and Fuller 2000). Collar, leg ring, subcutaneous anchor, breast and beak attachment systems have also been employed, albeit much less frequently and with varying levels of efficacy (Kenward 2000; Barron, Brawn, and Weatherhead 2010). For species intolerant of external devices, abdominal or subcutaneous implants are an option but require highly invasive surgical procedures (Barron, Brawn, and Weatherhead 2010; Boitani and Fuller 2000).

*Operational and ethical considerations*

Even with shrinking, ever more efficient and complex devices, researchers still face the balancing act of optimising data collection with appropriate attachment method and available power, linked to battery capacity, weight and the expected life of tags (Kays et al. 2015). Modern tracking systems make use of a variety of technologies to enhance power management such as solar re-charging (although this adds weight) and reprogramming of GPS, GSM and battery settings during deployment (Lopez-Lopez 2016; Tomkiewicz et al. 2010). The often-huge data sets derived from these technologies has generated the need for dedicated repositories such as [Movebank](file:///C:\Users\m290587\Downloads\Movebank) hosted by the [Max Planck Institute](https://www.ab.mpg.de/) and the [Seabird Tracking Database](file:///C:\Users\m290587\Downloads\Case%20Study%20Summary.png) managed by [BirdLife International](https://www.birdlife.org/) . Tracking data structures are not at all straightforward either, where spatial and temporal autocorrelations are endemic, making hypothesis testing very challenging (Fieberg et al. 2010; Noonan et al. 2019). There is help at hand for researchers though, via a myriad of readily available open-source statistical, GIS packages and dedicated on-line media (Gurarie et al. 2016).

In accordance with published literature, accepted best practice and some national licensing requirements, the combined tag and harness weight should not exceed 3-5% of individual body mass to avoid any deleterious effects on birds (Barron, Brawn, and Weatherhead 2010; Kenward 2000). However, Bodey *et al* (2018) suggest that these thresholds may be optimistic, finding negative effects upon survival, reproduction and parental care for tags weighing >1% body mass (Bodey et al. 2018). Their study didn’t necessarily advocate 1% as a new standard threshold (although desirable), rather that researchers give due prior consideration to the potential negative impacts of fitting tracking devices, not only to the subjects themselves but also to inherent bias in the derived data (Bodey et al. 2018; Kays et al. 2015).

**Biotelemetry in ecological study**

In the following section, we describe the benefits of biotelemetry in ecological study and explore the relevance to conservation translocations, structured around the themes of spatial behaviour, physio-social ecology, demography and human-wildlife interactions. We also touch upon the potential negative effects of deploying tracking devices.

*Spatial behaviour*

A staple of ecological research is understanding how an individual or population uses their landscape (Jonsson et al. 2016). Biotelemetry and automated analyses now make it relatively easy to precisely map foraging activity at multiple scales and habitat selection alongside macro-level movements such as dispersal patterns and migratory routes (Kays et al. 2015). Such data can reveal sex, age and seasonal differences in behaviour and migrant versus resident strategies (Buchan et al. 2019; Schlaich 2019). Furthermore, biotelemetry combined with remoted-sensed environmental data can pinpoint geographic and meteorological corridors and barriers to dispersal, migration and reproduction (Dobrynin et al. 2017; Schlaich 2019). These parameters are fundamental to understanding the drivers of dispersal patterns and establishment of translocated populations (Ewen and Armstrong 2007). To illustrate, Hirzel *et al* 2004 surprisingly found that initial distribution of reintroduced bearded vultures *Gypaetus barbatus* across the European Alps was strongly linked to underlying geology, suggesting that population recovery would be aided by concentrating releases within large limestone massifs (Hirzel et al. 2004).

Analyses of tri-axial acceleration, via machine-learning algorithms or high-resolution GPS data using multi-state Markov models, can classify behavioural modes and favoured sites at scales of just a few tens of meters (Nathan et al. 2012; Gurarie et al. 2016). Such detailed measurements are revolutionising our knowledge of animal foraging scales, niche separation and individual hunting strategies (Cumming, Henry, and Reynolds 2017). Other ecologically important locations such as nests and roosts, individual habitat specialisations and residence times, can also be readily identified by tracking data and open-source analytical packages (Bracis, Bildstein, and Mueller 2018). It has been documented that detailed knowledge of taxon-specific ecology, especially habitat selection, is key to successful translocations and biotelemetry has a clear role in provision of such insights (Ewen and Armstrong 2007; Bubac et al. 2019). Berger-Tal and Saltz (2014) specifically highlight the value to translocation success of examining movement behaviour, recommending a post-release monitoring framework measuring site fidelity, recurrent locations, conspecific proximity and individual variation (Berger-Tal and Saltz 2014).

*Physio-social ecology*

Telemetered data sheds new light on the physiological and cognitive processes influencing individual behaviour, personality and decision-making (Cagnacci et al. 2010; Nathan et al. 2012). Studies linking covariates of tracking data with remote-sensed mapping (such as primary productivity, vegetation and weather patterns) show considerable plasticity in animals’ responses to changing environmental conditions (Lopez-Lopez 2016; Kays et al. 2015). A recent multi-taxa meta-analysis of strategies within partial-migrant populations indicated a tendency towards residency due to increased exposure to anthropogenic threats faced by migrating individuals (Buchan et al. 2019). Plasticity in behaviour is a material consideration in planning translocations, where for example, taxon flexibility in habitat selection could allow for a broader range of suitable release sites and also facilitate environmental manipulation to enhance population recovery (Ewen and Armstrong 2007).

Overall Dynamic Body Acceleration (ODBA) derived from acceleration data can be used as a proxy for metabolic rate and energy expenditure, also illustrating the beneficial interplay of biomechanics and physiology within eco-behavioural research (Nathan et al. 2012; Wilson et al. 2006). Prior knowledge of taxon energetics can be informative for translocation management, for example in determining the need for food supplementation or targeted habitat enhancement (Armstrong et al. 2002). While most research has focussed on individuals, multi-tag studies can demonstrate population and community interactions, revealing the kinematics and determinants of collective, social and competitive behaviour (Cumming, Henry, and Reynolds 2017). For translocations, fitting multiple tracking devices can shed light on social interactions of released and wild conspecifics to reveal determinants of dispersal behaviour and help identify individual dispositions that underlie and modulate the expression of behaviour (McDougall et al. 2006; Kays et al. 2015). Specifically understanding the roles of personality and social structure allows managers to optimize the composition of release cohorts over time to increase reintroduction success (Berger-Tal and Saltz 2014).

*Demography*

A fundamental of ecological study and management, particularly pertinent to translocation planning and monitoring, is the ability to identify the demographic factors at play in population regulation (Cagnacci et al. 2010; Klaassen et al. 2014). Biotelemetry helps quantify vital rates such as survival and reproduction linked to sex, age along with important information on where and when they are expressed. Tags can also identify potential drivers of demography, such as habitat quality and compile distances and patterns of natal and breeding dispersal (Cagnacci et al. 2010). Modelling such parameters can provide highly informative predictions of viability and dynamics of translocated populations under different management scenarios (Ewen and Armstrong 2007). It is important to recognise that tracking devices almost certainly exert an influence on vital rates and this bias must be accounted for in any demographic modelling (Bodey et al. 2018). Arguably there are also welfare benefits derived from the long working life of modern tags and improved attachment methods, negating the need for subjects to incur recapture stress, but these must be weighed against the potentially negative effects associated with deployment (Peniche et al. 2011; Barron, Brawn, and Weatherhead 2010).

*Human-wildlife interactions*

Pinpointing mortality risk points and likely vectors for disease transmission is crucial for translocation success (Hays 2014; Carter et al. 2017). A prime case is the hen harrier *Circus cyaneus* in the UK, where an ongoing programme of satellite tagging has highlighted the geography of illegal persecution (Murgatroyd et al. 2019). Biotelemetry can reveal anthropogenic features such as highways, power distribution lines and landfill sites that act as both ecological attractors and traps (Buechley et al. 2018). More positively, tracking data can also help mitigate other ‘hotspots’ of potential human-wildlife conflict, such as helping guide the siting of wind farms and ‘green infrastructure’ in urban development plans, as well as direct the choice of culturally acceptable translocation locations (Lopez-Lopez 2016). Moreover, well publicised animal tracking has the capacity to engage and generate public interest above and beyond that of traditional media, which ultimately can drive far-reaching benefits for translocations, rewilding and wider environmental protection (Kays et al. 2015).

**References**

Armstrong, Doug P., R. Scott Davidson, Wendy J. Dimond, John K. Perrott, Isabel Castro, John G. Ewen, Richard Griffiths, and Jason Taylor. 2002. 'Population dynamics of reintroduced forest birds on New Zealand islands', *Journal of Biogeography*, 29: 609-21.

Barron, Douglas G., Jeffrey D. Brawn, and Patrick J. Weatherhead. 2010. 'Meta-analysis of transmitter effects on avian behaviour and ecology', *Methods in Ecology and Evolution*, 1: 180-87.

Berger-Tal, O., and D. Saltz. 2014. 'Using the movement patterns of reintroduced animals to improve reintroduction success', *Current Zoology*, 60: 515-26.

Bodey, Thomas W., Ian R. Cleasby, Fraser Bell, Nicole Parr, Anthony Schultz, Stephen C. Votier, and Stuart Bearhop. 2018. 'A phylogenetically controlled meta-analysis of biologging device effects on birds: Deleterious effects and a call for more standardized reporting of study data', *Methods in Ecology and Evolution*, 9: 946-55.

Boitani, L., and T. Fuller. 2000. *Research Techniques in Animal Ecology: Controversies and Consequences* (Columbia University Press).

Bracis, C., K. L. Bildstein, and T. Mueller. 2018. 'Revisitation analysis uncovers spatio-temporal patterns in animal movement data', *Ecography*, 41: 1801-11.

Bubac, Christine M., Amy C. Johnson, Janay A. Fox, and Catherine I. Cullingham. 2019. 'Conservation translocations and post-release monitoring: Identifying trends in failures, biases, and challenges from around the world', *Biological Conservation*, 238: 108239.

Buchan, C., J. J. Gilroy, I. Catry, and A. M. A. Franco. 2019. 'Fitness consequences of different migratory strategies in partially migratory populations: A multi-taxa meta-analysis', *J Anim Ecol*.

Buechley, E. R., M. J. McGrady, E. Coban, and C. H. Sekercioglu. 2018. 'Satellite tracking a wide-ranging endangered vulture species to target conservation actions in the Middle East and East Africa', *Biodiversity and Conservation*, 27: 2293-310.

Cagnacci, F., L. Boitani, R. A. Powell, and M. S. Boyce. 2010. 'Animal ecology meets GPS-based radiotelemetry: a perfect storm of opportunities and challenges', *Philosophical Transactions of the Royal Society B-Biological Sciences*, 365: 2157-62.

Carter, I., K. Walsh, J. Curson, A. W. Sainsbury, J. G. Ewen, and H. Matthew. 2017. 'Health and Disease in Translocated Wild Animals', *Ecohealth*, 14: S5-S6.

Cumming, G. S., D. A. W. Henry, and C. Reynolds. 2017. 'A framework for testing assumptions about foraging scales, body mass, and niche separation using telemetry data', *Ecology and Evolution*, 7: 5276-84.

Dobrynin, D. V., V. V. Rozhnov, A. A. Saveliev, O. V. Sukhova, and A. A. Yachmennikova. 2017. 'Integration of Satellite Tracking Data and Satellite Images for Detailed Characteristics of Wildlife Habitats', *Izvestiya Atmospheric and Oceanic Physics*, 53: 1060-71.

Ewen, J. G., and D. P. Armstrong. 2007. 'Strategic monitoring of reintroductions in ecological restoration programmes', *Ecoscience*, 14: 401-09.

Fieberg, J., J. Matthiopoulos, M. Hebblewhite, M. S. Boyce, and J. L. Frair. 2010. 'Correlation and studies of habitat selection: problem, red herring or opportunity?', *Philosophical Transactions of the Royal Society B-Biological Sciences*, 365: 2233-44.

Gurarie, E., C. Bracis, M. Delgado, T. D. Meckley, I. Kojola, and C. M. Wagner. 2016. 'What is the animal doing? Tools for exploring behavioural structure in animal movements', *Journal of Animal Ecology*, 85: 69-84.

Hays, G. C. 2014. 'Tracking animals to their death', *Journal of Animal Ecology*, 83: 5-6.

Hirzel, A. H., B. Posse, P. A. Oggier, Y. Crettenand, C. Glenz, and R. Arlettaz. 2004. 'Ecological requirements of reintroduced species and the implications for release policy: the case of the bearded vulture', *Journal of Applied Ecology*, 41: 1103-16.

Hofman, M. P. G., M. W. Hayward, M. Heim, P. Marchand, C. M. Rolandsen, J. Mattisson, F. Urbano, M. Heurich, A. Mysterud, J. Melzheimer, N. Morellet, U. Voigt, B. L. Allen, B. Gehr, C. Rouco, W. Ullmann, O. Holand, N. H. Jorgensen, G. Steinheim, F. Cagnacci, M. Kroeschel, P. Kaczensky, B. Buuveibaatar, J. C. Payne, I. Palmegiani, K. Jerina, P. Kjellander, O. Johansson, S. LaPoint, R. Bayrakcismith, J. D. C. Linnell, M. Zaccaroni, M. L. S. Jorge, J. E. F. Oshima, A. Songhurst, C. Fischer, R. T. Mc Bride, J. J. Thompson, S. Streif, R. Sandfort, C. Bonenfant, M. Drouilly, M. Klapproth, D. Zinner, R. Yarnell, A. Stronza, L. Wilmott, E. Meisingset, M. Thaker, A. T. Vanak, S. Nicoloso, R. Graeber, S. Said, M. R. Boudreau, A. Devlin, R. Hoogesteijn, J. A. May, J. C. Nifong, J. Odden, H. B. Quigley, F. Tortato, D. M. Parker, A. Caso, J. Perrine, C. Tellaeche, F. Zieba, T. Zwijacz-Kozica, C. L. Appel, I. Axsom, W. T. Bean, B. Cristescu, S. Periquet, K. J. Teichman, S. Karpanty, A. Licoppe, V. Menges, K. Black, T. L. Scheppers, S. C. Schai-Braun, F. C. Azevedo, F. G. Lemos, A. Payne, L. H. Swanepoel, B. V. Weckworth, A. Berger, A. Bertassoni, G. McCulloch, P. Sustr, V. Athreya, D. Bockmuhl, J. Casaer, A. Ekori, D. Melovski, C. Richard-Hansen, D. van de Vyver, R. Reyna-Hurtado, E. Robardet, N. Selva, A. Sergiel, M. S. Farhadinia, P. Sunde, R. Portas, H. Ambarli, R. Berzins, P. M. Kappeler, G. K. Mann, L. Pyritz, C. Bissett, T. Grant, R. Steinmetz, L. Swedell, R. J. Welch, D. Armenteras, O. R. Bidder, T. M. Gonzalez, A. Rosenblatt, S. Kachel, and N. Balkenhol. 2019. 'Right on track? Performance of satellite telemetry in terrestrial wildlife research', *PLoS One*, 14.

Jonsson, K. A., A. P. Tottrup, M. K. Borregaard, S. A. Keith, C. Rahbek, and K. Thorup. 2016. 'Tracking Animal Dispersal: From Individual Movement to Community Assembly and Global Range Dynamics', *Trends Ecol Evol*, 31: 204-14.

Kays, R., M. C. Crofoot, W. Jetz, and M. Wikelski. 2015. 'Terrestrial animal tracking as an eye on life and planet', *Science*, 348: 10.

Kenward, R.E. 2000. *A Manual for Wildlife Radio Tagging* (Elsevier Science).

Klaassen, R. H. G., M. Hake, R. Strandberg, B. Koks, C. Trierweiler, K. M. Exo, F. Bairlein, and T. Alerstam. 2014. 'When and where does mortality occur in migratory birds? Direct evidence from long- term satellite tracking of raptors', *Journal of Animal Ecology*, 83: 176-84.

Lopez-Lopez, P. 2016. 'Individual-based tracking systems in ornithology: welcome to the era of big data', *Ardeola-International Journal of Ornithology*, 63: 103-36.

Mattern, Thomas, Juan F. Masello, Ursula Ellenberg, and Petra Quillfeldt. 2015. 'Actave.net – a web-based tool for the analysis of seabird activity patterns from saltwater immersion geolocators', *Methods in Ecology and Evolution*, 6: 859-64.

McDougall, P. T., D. Réale, D. Sol, and S. M. Reader. 2006. 'Wildlife conservation and animal temperament: causes and consequences of evolutionary change for captive, reintroduced, and wild populations', *Animal Conservation*, 9: 39-48.

Murgatroyd, M., S. M. Redpath, S. G. Murphy, D. J. T. Douglas, R. Saunders, and A. Amar. 2019. 'Patterns of satellite tagged hen harrier disappearances suggest widespread illegal killing on British grouse moors', *Nat Commun*, 10: 1094.

Nathan, R., O. Spiegel, S. Fortmann-Roe, R. Harel, M. Wikelski, and W. M. Getz. 2012. 'Using tri-axial acceleration data to identify behavioral modes of free-ranging animals: general concepts and tools illustrated for griffon vultures', *J Exp Biol*, 215: 986-96.

Noonan, Michael J., Marlee A. Tucker, Christen H. Fleming, Thomas S. Akre, Susan C. Alberts, Abdullahi H. Ali, Jeanne Altmann, Pamela Castro Antunes, Jerrold L. Belant, Dean Beyer, Niels Blaum, Katrin Böhning-Gaese, Laury Cullen Jr., Rogerio Cunha de Paula, Jasja Dekker, Jonathan Drescher-Lehman, Nina Farwig, Claudia Fichtel, Christina Fischer, Adam T. Ford, Jacob R. Goheen, René Janssen, Florian Jeltsch, Matthew Kauffman, Peter M. Kappeler, Flávia Koch, Scott LaPoint, A. Catherine Markham, Emilia Patricia Medici, Ronaldo G. Morato, Ran Nathan, Luiz Gustavo R. Oliveira-Santos, Kirk A. Olson, Bruce D. Patterson, Agustin Paviolo, Emiliano Esterci Ramalho, Sascha Rösner, Dana G. Schabo, Nuria Selva, Agnieszka Sergiel, Marina Xavier da Silva, Orr Spiegel, Peter Thompson, Wiebke Ullmann, Filip Zięba, Tomasz Zwijacz-Kozica, William F. Fagan, Thomas Mueller, and Justin M. Calabrese. 2019. 'A comprehensive analysis of autocorrelation and bias in home range estimation', *Ecological Monographs*, 89: e01344.

Peniche, G., R. Vaughan-Higgins, I. Carter, A. Pocknell, D. Simpson, and A. Sainsbury. 2011. 'Long-term health effects of harness-mounted radio transmitters in red kites (Milvus milvus) in England', *Vet Rec*, 169: 311.

Schlaich, A. E. 2019. 'Migrants in Double Jeapardy', *PhD Thesis*.

Taylor, Philip D., Tara L. Crewe, Stuart A. Mackenzie, Denis Lepage, Yves Aubry, Zoe Crysler, George Finney, Charles M. Francis, Christopher G. Guglielmo, Diana J. Hamilton, Rebecca L. Holberton, Pamela H. Loring, Greg W. Mitchell, D. Ryan Norris, Julie Paquet, Robert A. Ronconi, Jennifer R. Smetzer, Paul A. Smith, Linda J. Welch, and Bradley K. Woodworth. 2017. 'The Motus Wildlife Tracking System: a collaborative research network to enhance the understanding of wildlife movement', *Avian Conservation and Ecology*, 12.

Tomkiewicz, S. M., M. R. Fuller, J. G. Kie, and K. K. Bates. 2010. 'Global positioning system and associated technologies in animal behaviour and ecological research', *Philosophical Transactions of the Royal Society B-Biological Sciences*, 365: 2163-76.

Whitford, Malachi, and A. Peter Klimley. 2019. 'An overview of behavioral, physiological, and environmental sensors used in animal biotelemetry and biologging studies', *Animal Biotelemetry*, 7: 26.

Williams, H. J., L. A. Taylor, S. Benhamou, A. I. Bijleveld, T. A. Clay, S. de Grissac, U. Demsar, H. M. English, N. Franconi, A. G?mez-Laich, R. C. Griffiths, W. P. Kay, J. M. Morales, J. R. Potts, K. F. Rogerson, C. Rutz, A. Spelt, A. M. Trevail, R. P. Wilson, and L. B?rger. 2020. 'Optimizing the use of biologgers for movement ecology research', *Journal of Animal Ecology*, 89: 186-206.

Wilson, R. P., E. L. C. Shepard, and N. Liebsch. 2008. 'Prying into the intimate details of animal lives: use of a daily diary on animals', *Endangered Species Research*, 4: 123-37.

Wilson, R. P., C. R. White, F. Quintana, L. G. Halsey, N. Liebsch, G. R. Martin, and P. J. Butler. 2006. 'Moving towards acceleration for estimates of activity-specific metabolic rate in free-living animals: the case of the cormorant', *Journal of Animal Ecology*, 75: 1081-90.
